# Handling of Fresh Vegetables: Knowledge, Hygienic Behavior of Vendors, Public Health in Maputo Markets, Mozambique

**DOI:** 10.3390/ijerph17176302

**Published:** 2020-08-29

**Authors:** Cátia Salamandane, Filipa Fonseca, Sónia Afonso, Maria Luisa Lobo, Francisco Antunes, Olga Matos

**Affiliations:** 1 Medical Parasitology Unit, Group of Opportunistic Protozoa/HIV and Other Protozoa, Global Health and Tropical Medicine, Instituto de Higiene e Medicina Tropical, Universidade Nova de Lisboa, 1700-001 Lisbon, Portugal; catialsilvamac@gmail.com (C.S.); luisalc@ihmt.unl.pt (M.L.L.); 2 Nova School of Business and Economics, Universidade Nova de Lisboa, Faculdade de Economia e Gestão, 1700-001 Lisbon, Portugal; 3 Faculdade de Ciências de Saúde (FCS), Universidade Lúrio, Nampula 4250, Mozambique; 4 Parasitology Department of Veterinary Faculty, Universidade Eduardo Mondlane, Maputo 3453, Mozambique; safonso9@hotmail.com; 5 Environmental Health Institute, Faculdade de Medicina da Universidade de Lisboa, 1700-001 Lisbon, Portugal; fantunes@medicina.ulisboa.pt

**Keywords:** fresh vegetables, gastrointestinal diseases, public-health, vendors, behavior, Maputo-Mozambique

## Abstract

In developing countries, markets are the main supply of horticultural products to populations, but this can pose a public health challenge due to the risk of the fecal-oral transmission of gut pathogens. This transmission is strongly associated with inadequate public sanitation or low standards of personal and domestic hygiene, and their prevalence can cause gastrointestinal diseases, which are the third leading cause of death in Mozambique. This study aims at assessing the risk for public health of horticultural products supply chain, from the farmers-vendors to the consumers, in municipal markets in Maputo-City, Mozambique. Surveys (75) were conducted on vendors and an observational analysis was performed in the markets under study. The results showed that 62% of the vendors had access to water from boreholes or artisanal sources and the issue “access to water” was significantly different between markets (*p* = 0.004). Of the vendors who wash their products (53.3%), only 7.5% use tap-water for this purpose, with the difference in attitudes being statistically significant between vendors in the markets (*p* = 0.035). The majority (60.4%) said that vegetables and fruits can cause diseases due to pesticides and only 31.3% believe that the diseases may be related to poor hygiene. Despite the vendors’ low knowledge of Good Hygiene Practices (GHP), we noticed that women have better practical assimilation of GHP when compared to men (*p* = 0.008). Although Maputo’s markets are struggling to achieve quality hygiene standards in a reliable and sustainable manner, their resources are limited and significantly different (*p* = 0.044) from market to market, and this problem remains a concern for the public-health authorities of the city. In conclusion, the provision of adequate drinking water and sewage disposal systems, together with education for health of vendors, can reduce the risk of contamination of fresh food by the more common organisms causing diarrhea in children, including intestinal parasites.

## 1. Introduction

In developing countries, the quality and safety of food are a continuous challenge [1]. The populations in urban developing areas can struggle with greater or lesser food shortages, while in sub-urban areas this problem does not usually arise, since agricultural production occurs outside the urban centers. However, the issue of “food safety” raises great concern both in urban and sub-urban areas. In the latter, despite the larger supply of food, there is no guarantee of quality in their supply systems [2]. The consumption of fresh foods, such as horticultural products, corresponds to a lifestyle recently adopted worldwide, in search of a healthy life; although it can be a challenge, due to contamination by microorganisms, which can be detected in this type of food and also in water [2]. In recent years, foodborne disease outbreaks have been strongly linked to pathogenic bacteria and parasites [3,4,5,6,7]. Likewise, the consumption of fruits and vegetables can be a paradox, because, on the one hand, it is advisable for better health, with several benefits, but on the other, it can be a gateway for pathogenic microorganisms and a source of chronic diseases, such as certain types of cancer [4,6,7]. In Mozambique, very low consumption of horticultural products has been reported—less than 5% intake per day of five or more daily servings of fruits and vegetables [6]—and it is a curious fact, because this is an agricultural country, where the habit of generating family income with agricultural products is important especially in rural areas. Contamination by microorganisms from fresh crops mainly green leafy vegetables and those growing close to the ground, such as lettuce, cabbage and tomatoes, is high when compared with other types of vegetables [8,9]. In many developing countries like Mozambique, municipal markets, formal or informal, are the main supply of horticultural products from farms located in peri-urban areas [9].

Hazard Analysis and Critical Control Points are not being regulated or frequently applied in any stage of handling or processing food, from the field to the vendor, and interfere with food safety, especially fresh food. Moreover, many gastrointestinal diseases, whose prevalence is strongly linked with the ingestion of pathogenic organisms in food and water contaminated by feces from humans or animals excreting those organisms, often associated with poor sanitation or low standards of personal and domestic hygiene [10]. In fact, this is the leading cause of gastrointestinal diseases, especially in children in developing countries, where 21% of children do not reach the age of five (about 2.5 millions of deaths per year) [11]. More specifically in Mozambique, gastrointestinal diseases are the third leading cause of death in infants and young children [12,13,14].

This study aims to assess the public health risk of the fresh-vegetables supply chain, from farmers to consumers, in the municipal markets of the city of Maputo, Mozambique. In addition, it is planned to emphasize operations such as production, distribution, transportation, storage and handling of fresh food by farmers and vendors and ultimately determine the degree of awareness of vendors on these issues.

## 2. Methodology

### 2.1. Study Area

**Figure 1:**
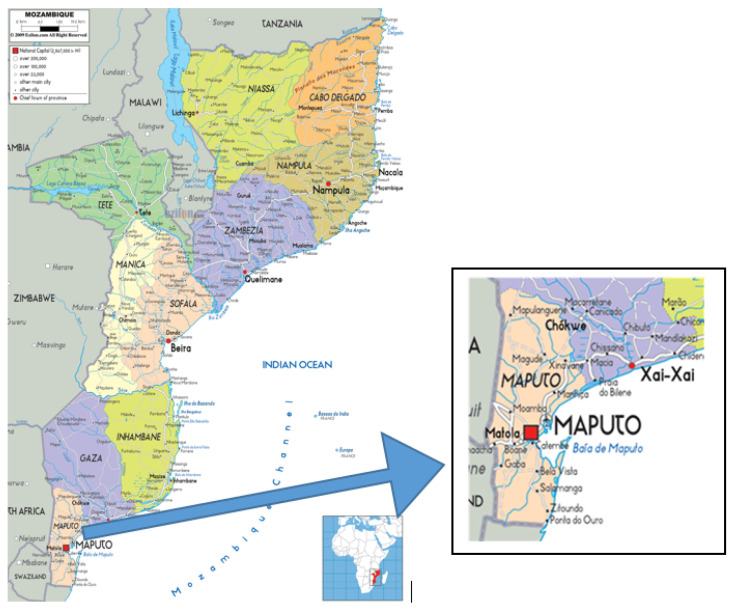
Maps of Mozambique and Maputo province. Source: https://images.search.yahoo.com/search/images?p=mozambique+map&fr=mcafee_uninternational&imgurl=https%3A%2F%2Fwww.nationsonline.org%2Fmaps%2FMozambique_map.jpg#id=2&iurl=http%3A%2F%2Fwww.ezilon.com%2Fmaps%2Fimages%2Fafrica%2Fpolitical-map-of-Mozambique.gif&action=click.

Maputo is the capital of Mozambique (Figure 1) and the city with the highest population density in the country. It has approximately 347.69 km^2^ of area and 3670.6/km^2^ of total population density and over time Maputo has been registering growth in population density, being a major concern for government entities including municipal services [12].

According to the last census, there are 27,909,798 people in Mozambique, where 33.4% are living in urban areas and 66.6% in the countryside [14]. In Maputo, we can find multicultural international identities—people from all over the world, the largest foreign community coming from Malawi and also from different national identities, from other provinces and rural areas [12,13,14].

**Figure 2:**
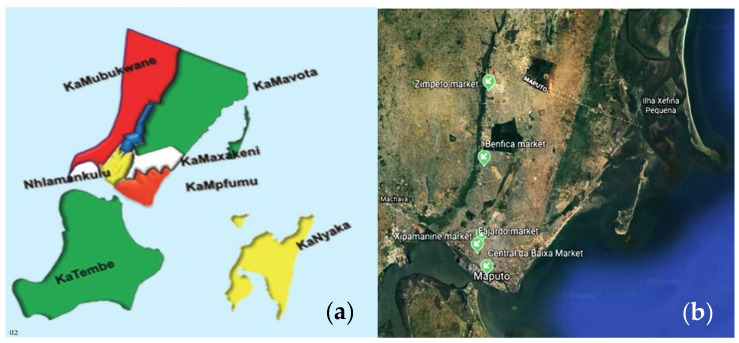
Sampling sites: (**a**) Map of municipal districts; (**b**) Markets sampled. *Source*: http://docplayer.com.br/12277474-Caderno-de-oportunidades-de-investimento-cidade-de-maputo.html.

Maputo City is divided into seven municipal districts (Figure 2a), namely KaMpfumo, Nlhamakulu, KaMaxakeni, KaMubukwana, KaMavota, KaTembe and KaNyaka, and is subdivided into 63 neighbourhoods. The most populous ones are KaMubukwana and KaMavota per Km^2^. The present study was carried out in three municipal districts, Kampfumo, Kamubukwana and Nlhamankulo, as illustrated in Figure 2. According to the City Council’s postulate for fairs and markets, five types of municipal markets (Figure 2b) were chosen: a wholesale, three retail markets and one production zone.

### 2.2. Maputo Municipal Markets

According to the City Council’s postulate for fairs and markets [15], there are four types of markets in the city of Maputo: the supply market, wholesale, neighborhood market and informal market. Despite this characterization, the first three markets are considered formal. They have legal activities, are well organized and structured, and the vendors are properly licensed, working for the city and thus profiting from the maintenance of the city services. On the other hand, the informal markets are those that appeared spontaneously, without a license from the City Council and with a new approach, selling on the streets, corners and outside the markets to respond to immediate needs, remaining for a short period of time [15,16,17,18]. Regardless of this, those informal vendors also pay fees to Maputo’s City Council. The informal sector is strictly linked to the formal sector, supporting each other, which reflects the weakness of government institutions, a consequence of the inadequate application of public policies and the frustrations of economic strategies. Sometimes the government encourages them in order to promote the growth of the private sector, even with the imbalances between these markets [16,17].

### 2.3. City Council

The entity responsible for the management of all the municipal markets in Maputo is the City Council. It is composed by nine advisory support units and 12 technical and administrative departments which are: Health and Social Action (PSAS), Education Culture and Sports (PECD), Finances (PF), Infrastructure Facilities (PIE), Planning and Urban Environment (PPUA), Human Resources (PRH), Markets and Fairs (PMF), Solid Waste Management and Salubrity (PGRSS), Cemeteries’ Management (PGC), Transport and Traffic (PTT), Economic Activities (PAE) and Information Systems (PSI) [15].

The main departments that have a direct intervention in the markets are the PMF, the PSAS, the National Inspection of Economic Activities (INAE) and Mozambique Red Cross (CVM). While the PMF is in the administrative sector, creating the regulations for markets operability, the Board of Directors is responsible for recruiting each market manager, and PSAS is mainly dealing with public health, promoting campaigns, training sessions close to focal groups and supervise actions to promote the quality of food, resources and health environment in the markets. They also promote a better social life. The newly created INAE is a State Institution, under the supervision of the Ministry of Commerce and Industry, which oversees all areas where there are industrial or commercial activities, or even providing services. They can prevent products from becoming outdated, product corruption and counterfeiting. CVM is present mainly in public health activities and campaigns of good hygiene and sanitation practices (GHP—the minimum sanitary and hygiene practices for food handlers to ensure that food is safe and suitable for human consumption) working directly with focus groups in the markets, in collaboration with PSAS.

### 2.4. Selection Criteria

After the City Council approval for the present study in Maputo markets, surveys were applied to vendors in five markets: Benfica and Zimpeto, corresponding to Kamubukwana municipal district, Fajardo and Xipamanine corresponding to Nlhamakulu, and Central da Baixa corresponding to Kampfumo municipal district. Those markets were chosen because of their demand and popularity in Maputo. The surveys were applied randomly only to vegetables vendors and according to the interviewee’s feasibility, without interfering with their ordinary workday. The surveys were applied in two phases: the first was the period corresponding to the dry season, from February to April 2019, and the second, to the rainy season, from August to October 2019. A total of 75 surveys were made.

For the observational analysis, at least two vendors were chosen in each market to monitor all the stages of the sales process—from product acquisition, transportation, sanitization, storage and finally the display of products for sale. The environmental sanitation, water sources and toilet conditions were also considered without interfering with the usual daily process of vendors and according to associated risk factors as described by Trafialek et al. [19].

### 2.5. Vendors Profile

The characterization of vendors results from small interviews and observational analysis in each market.

Of a total of 75 vendors from five open markets interviewed, 86.7% were women and 13.3% men. About 51.7% of the vendors were between 18 and 45 years old and 46.9% were over 45 years old. Only 2.7% of those vendors were under 18 years old. As for education level, 45.3% of respondents indicated that they had completed elementary school and 41.3% the secondary school; only 13.3% were illiterate. As for the time spent in the activity of vendors, 65.3% said they had been selling vegetables in markets for less than 10 years and 34.7% for more than 10 years. The characteristics of the vendors of the five markets studied are summarized in Table 1.

### 2.6. Statistical Analysis

For the present study, IBM SPSS (IBM Corp, NY, USA) Statistics 26 package was used to analyze the obtained results. Markets were compared with each other considering variables that may represent risk for public health, see Table S1 in the Supplementary Materials. Chi-square or Fisher tests were used. Fisher’s test was used alternatively whenever the 20% of cells with expected values of count (frequency) were less than five (5). Multiple regression was also used to analyze the relations between vendors profile and their impact on vendors’ behavioral attitudes, and the distribution and availability of tap water and the presence of toilets among the studied markets, according to the main institution responsible for resource management.

## 3. Results

### 3.1. Products Acquisition

Some of the horticultural products sold in the markets are from peri-urban zones (30.7%), acquired directly from the farmers’ areas, mostly from “zonas verdes/green zones*”. These products also came from wholesale markets (44%) and other markets (10.7%) where products are resold in small quantities, e.g., in Fajardo. The remaining vendors (14.7%) bring their own products from other production areas, outside the city of Maputo, such as Chokwé, Manhiça, Marracuene, Chibuto, Zavala, Boane, Bobole and others (Table 2).

### 3.2. Water Sources for Irrigation in Production Fields

**Figure 3:**
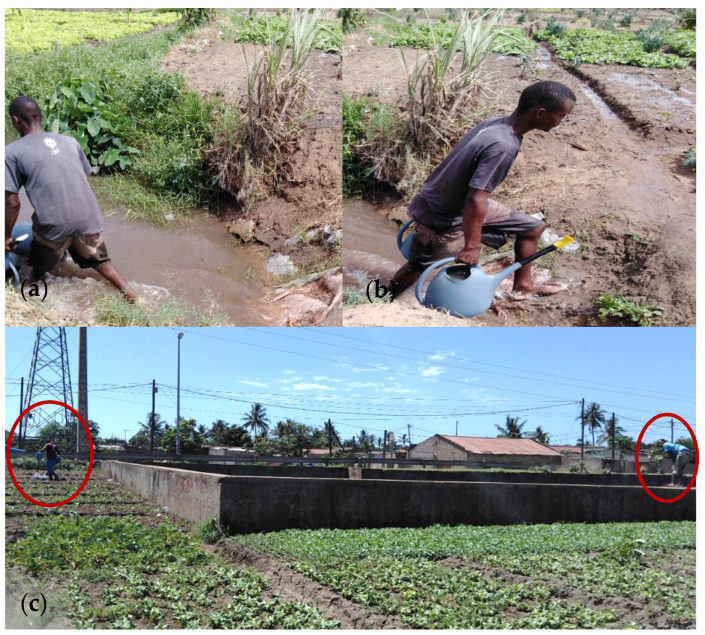
Farmers collecting water: (**a**) a man collecting water from artisanal well; (**b**) carrying it for irrigation in Infulene area; (**c**) manual crops irrigation (men at left) and water collection for irrigation, in a section of drainage ditch in the city of Maputo.

After observational analysis, on the production fields, it was possible to understand the main source of water for irrigation. In the Infulene valley—one of the most important producing fields located in a plain rift in the peri-urban area of the city of Maputo, water is supplied by the Mulaúze River (tributary of the Infulene River). However, the Infulene valley comprises an area between a brewery and drainage ditches, where urban sewage is dumped. Farmers build artisanal water wells in their farms to ensure water for irrigation. In addition, they also collect water from drainage ditches to ensure daily irrigation (Figure 3). After the products are harvested, they are washed with this water, this being the first step in cleaning the vegetables.

### 3.3. Packaging, Transport and Storage

**Figure 4:**
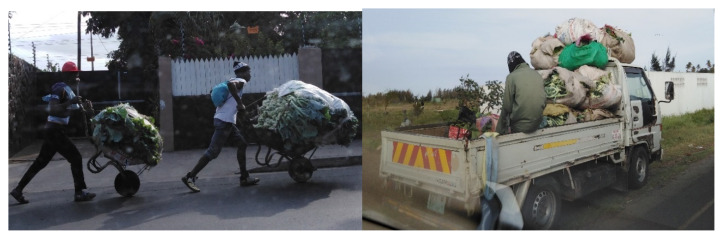
Transportation of products to markets, before sale, in wheelbarrows and propylene raffia bags.

Based on vendors’ speech and from an observational perspective, we found that, in general, the transport is performed using pick-up trucks (58.7%) or public transportation (6.7%) or even by wheelbarrows, depending on the distance between the place where the products are purchased and the place where they are sold (Figure 4 and Table S1 in the Supplementary Materials). Once in the market, the vendors start by cleaning their place of sale or stand and usually store vegetables under different security risk conditions.

**Figure 5:**
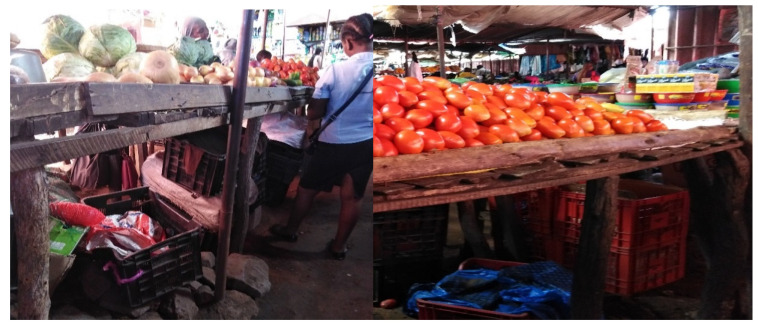
Storage of products below their stands at market in plastic bags and plastic boxes on Fajardo’s market.

During observational analysis, only 9.3% of the vendors, from Zimpeto (8%) and Central markets (1.3%), said that they transport products in suitable boxes. About 7% of vendors store their products in straw baskets and 84% in propylene raffia bags (Figure 4 and Figure 5).

In the central market, about 100% of the interviewees said they store their products below their stands in the market. On the other hand, in the Fajardo market, 21.4% prefer to keep their products on the market behind the stands and another 21.4% prefer to take the products home. The majority (42.9%) prefer to keep the products in private warehouses outside the market, paying daily fees and the remaining vendors (14.3%) prefer to store their products in a private warehouse within the market (Table S1 on Supplementary Materials).

In the Zimpeto and Xipamanine markets, similar percentages, 42.1% and 41.7%, were registered for those who prefer to keep their products in private warehouses in the market. Nevertheless, in Zimpeto market, none of the vendors keeps their products in a warehouse outside the market and 26.3% take the products home or keep them at their stand (31.6%). In Benfica market, vendors are more confident to keep their products on private warehouses outside the market (45%) than inside the market (10%), but few of them still take the chance of keeping the products below their stands (25%), while the remaining (20%) prefer not to risk it and take their products home until the next day. In general, the global percentage indicates that most of the vendors in the markets prefer to keep their products in the market below their stands (38.7%) and the minority prefer to take them home (17.3%).

The results of the answers given to “*how do you transport the products*” showed that there is a significant difference (*p* = 0.014) between markets on their methods for products transportation and it can make a difference on the product quality until it reaches the final consumer. In this case, the vendors of the Fajardo market were the ones with the worst method of transporting their products, using mostly pick-up-track with public transport. On the other hand, the vendors in the Benfica market were the ones who best transported their products, using the pick-up-track more frequently for the exclusive transport of vegetables.

To better understand the vendors’ attitudes towards transportation, displays and products storage processes, we analyzed their profile variables and the variables that may represent their knowledge on food safety on the questions “*do you think vegetables can cause disease*?”, “*why do you think vegetables can cause disease*?” and we observed that there were no significant differences (*p* = 0.228) for ANOVA test nor for any of the other independent variables (vendor’s profile). We also observed that the variable “education” was not a predictor for greater knowledge on food safety when compared with other independent variables (*p* = 0.053).

### 3.4. Knowledge and Attitude: Sanitization and Display Conditions in the Markets

Hygiene practices depend on the daily resources and the perception of each vendor about food safety. Most of the markets do not have access to tap water, but to a well. Others have none of these resources; therefore, as an alternative, they buy water near the markets, at schools or in the vicinity. However, a downtown market supplies tap water every day.

Regarding to products behavior, 46.7% of the vendors do not wash the vegetables before selling them. Of those who wash their products before selling, only 7.5% use tap water, while the other 92.5% wash them in plastic containers using untreated water.

**Figure 6:**
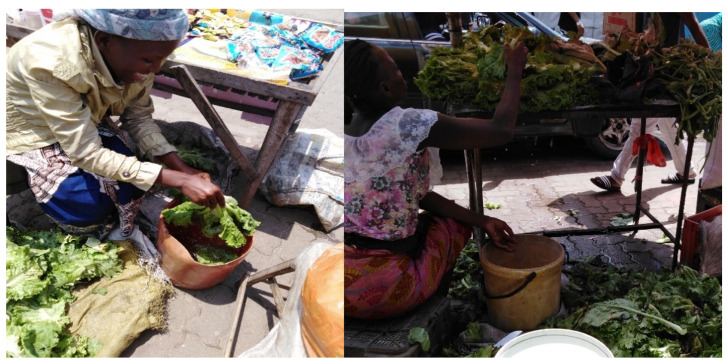
Handling of lettuce and cabbage in a plastic container, on Benfica’s market.

The behavioral process in general depends on each market and according to its resources. In the central market, most vendors (87.5%) wash their products with tap water every day. The Benfica and Xipamanine markets have a high rate of washing habits before showing their products for sale—70% and 66.7%, respectively. However, in these two markets, the vendors wash their products without a sanitizing solution within the same non-sanitized container (Figure 6).

The lowest rates of washing horticultural products were found in the Fajardo and Zimpeto markets—28.6% and 31.6%, respectively.

**Figure 7:**
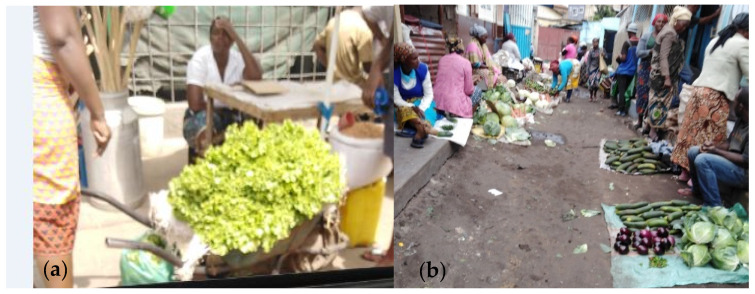
Products displays: (**a**) Lettuce in a wheelbarrow; (**b**) other vegetables inside or over propylene raffia bags on the floor on Fajardo’s market.

With regard to the display of products, it became clear that all the markets studied, excluding the Central, sell the products directly in “tchova” (a typical wheelbarrow, Figure 7a) or inside or over a propylene bag on the floor (Figure 7b) or even at a stand of handcrafted wooden stakes.

**Figure 8:**
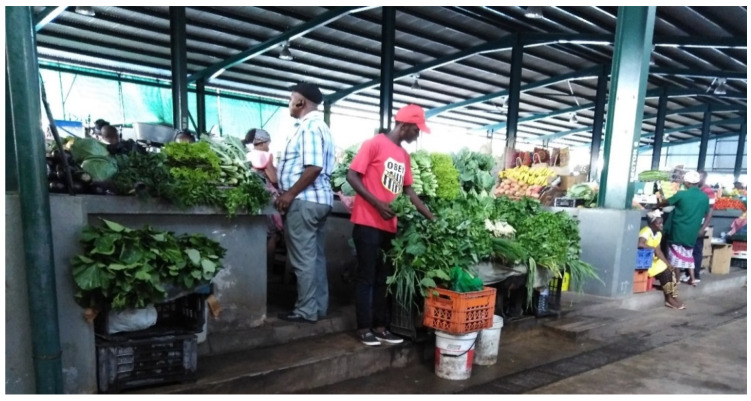
Stands at Central market sufficiently distant from the floor.

The case is clearly different in the Central market, because it has another type of resources and the vendors maintain its good structure (Figure 8). Their stands are separated from the floor at least 1m to prevent the contact of the products with the floor.

After an evaluation of results to analyze if there was a relation between any of independent predictors variables, such as age, gender and education, (vendor’s profile) and their behavioral attitudes on each variables considering the questions “do you *wash products before sale*”, “*How do you wash theme*”, we observed that, in all markets, women were the ones who had the best practices concerning the sanitization of the vegetables for sale in comparison with men, with a significant *p* value (*p* = 0.008) (Table 3).

### 3.5. Market Conditions: Water Resources for Behavioral

When vendors were asked about the water sources used in the markets, 13.3% said that water within the markets is provided by the water supply system—*Fundo de Investimento e Património do Abastecimento de Água* (FIPAG), 62.6% referred to the borehole or artisanal waterhole, while 24% said that there is no water in the markets and they need to search for it in the vicinity. As for the knowledge of disease transmission through the consumption of vegetables, 64% said that these products could transmit diseases. However, of those, only 31.3% associated this problem to the lack of hygiene and 60.4% answered that this can happen when people do not respect the pesticide application period.

### 3.6. Market Conditions: Toilets Conditions

In general, it was possible to find toilets in all the markets, from public to private. The access to the toilets is granted by paying a symbolic fee to help in their daily maintenance. Public toilets from the City Council are present in most markets (82.7%). On the other hand, the private toilets (14.7%) are usually outside the markets under private management. Although there is a preference for public toilets at the markets, there is no difference in terms of conditions between those two types. When queried about the access to water in the toilets, 4% of vendors said “*no*” and 8% said “*sometimes*”. Most vendors (53.3%) said that toilets had water available in recipients (plastic buckets and gallons) for general use, and the remaining vendors (34.7%) said that there was tap water.

### 3.7. Market Conditions: Solid Waste Management in Markets

According to municipal entities, the cleaning of the markets is the vendors’ entire responsibility in each vending stand, but the Municipal City Council is responsible for the waste collection all over the city, via the PGRSS department [18,20]. For markets and fairs, the PSAS has designated large containers to deposit their waste, that later will be transported to the municipal disposal site [21]. Garbage containers were seen in all the markets visited. These markets have a scheduled day a week that vendors use to collect trash and clean the market according to market rules before opening to the public.

**Figure 9:**
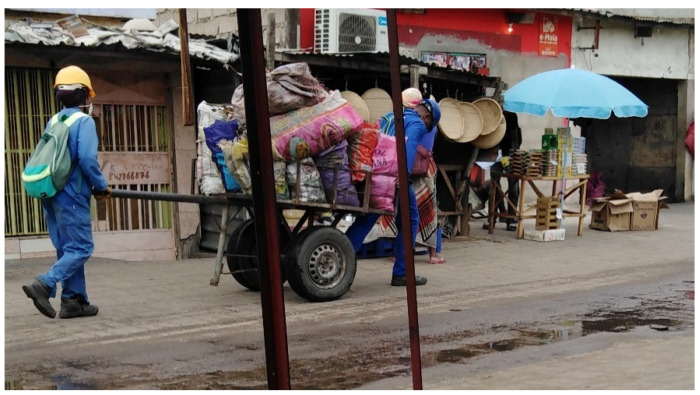
Garbage collection inside Benfica’s market to dump in the large containers outside the market, before opening.

Most vendors (82.7%) prefer to fill in the trash bag after cleaning and throw it away in the garbage container at the end of the day, and 14.7% prefer to throw the trash in the garbage containers immediately after the cleaning. In the central market, 60% of respondents said they keep their garbage in bags for disposal at the end of the day, as well as 64.3% in the Fajardo market. Although the majority of respondents in the Xipamanine market claim that waste management is individual, as in the Benfica and Zimpeto markets, the majority—100%, 90% and 89.5%, respectively—prefer to dump the garbage at the end of the day. All markets throw garbage in containers before opening or immediately after opening (Figure 9). The vendors’ attitudes are summarized in Table S1 attached in the Supplementary Materials.

### 3.8. Markets Management

The City Council of Maputo is the higher entity responsible for the management of markets and fairs through the PMF department. After a short interview with a team from this department, they said that it is difficult to work with vendors because of their resistance to changes. For example, the PMF trained the vendors of the new fish market of Maputo but realized that they are averse to applying the new concepts of hygiene and quality that should exist in a high-quality market.

The PMF team also stated that some problems are difficult to solve because the vendors do not agree that water should be supplied, and its quality controlled daily in the market (the fish market, for example). For fresh food markets, vendors activities and supervision as well as their training have been taken by the Health Action Social Department (PSAS). This department mainly manages issues in the area of public health, having direct intervention in the markets through focus groups in each market. This department collaborates mostly with the Food Hygiene Centre (CHA), CVM and PGRSS. They train focus groups on good hygiene practices (GHP) and good agricultural practices (GAP) and these groups spread information to other vendors in the market. In addition to training, they supervise the facilities. With the recent creation of INAE in 2009, conflicts have arisen based on the supervision carried out by both institutions, INAE and PSAS. The supervision of the markets shows that some improvements are still difficult, whether in terms of legal knowledge or in terms of training carried out by their technical teams.

With INAE, we noted that while there is a clear view of the surveillance of industrial and commercial activities to ensure quality services to the consumer and prevent corruption, there is a gap in its technical team regarding the application of their actions. After gaining experience, it has been observed that they begin to cooperate simultaneously with other institutions to do jobs other than those for which they were trained.

The general operating conditions of the markets are available differently in each market. Their operating conditions also depend on the market’s location. All the markets are managed by the same institution (PMF), that is responsible for the development of GHP facilities. We analyzed two potential important issues, concerning management resources, namely “*is there any toilet near*?” and “*is there water*? *how is it available*?” to evaluate if these facilities are uniformly distributed among the studied markets.

The results showed that there are statistically significant differences in the availability distribution of the evaluated resources in the studied markets (*p* = 0.044). Moreover, this difference is more pronounced for the variable “*is there water? how is it available?*” *(p* = 0.021) (Table 3). Although all markets belong to the same administrative institution (PMF), the distribution of the assessed resources is not uniformly available in all of them, the most privileged being those located in the city center (see Table S1 in the Supplementary Materials).

## 4. Discussion

This study aimed to assess the public health risk of the horticultural products, especially fresh-vegetables supply chain, from farmers to consumers, in the municipal markets of the city of Maputo, Mozambique. Special importance was given to the areas of production, distribution, transport, storage and handling of fresh vegetable by the vendors; there was a concern to determine the degree of awareness of vegetable farmers in relation to these problems.

The Infulene valley is one of the two largest agricultural areas around the city of Maputo, where most leafy vegetables are produced. This agricultural valley is provided by the drainage ditch of the Infulene River. In this region, through an observational analysis, it was possible to understand that the irrigation water comes from a nearby underground spring, which supplies water to the Infulene valley. Unfortunately, this valley has been contaminated with urban sewage from Infulene’s wastewater treatment plant (WWTP). These waters have not been treated as recommended, as reported by Matangue et al. [8], who showed that these irrigation waters were contaminated with pathogenic bacteria and *Giardia* spp. Cambaza et al. [22] reported the occurrence of high levels of fecal coliforms and *Escherichia coli* in lettuce sold in Maputo markets, probably from this producing area. Some authors emphasize that wastewater discharged from farms cannot be neglected because they are considered as an important source of water pollution, including intestinal pathogens (e.g., *E. coli*, *G. duodenalis* and *Cryptosporidium* spp. [23]. However, if we can control the pollution associated with wastewater discharged daily in WWTP-Infulene near the vegetable fields and untreated water collected in the valley, we can avoid a worse scenario for these horticultural products. Although most farmers have said that they know how to treat water and that the local WWTP is from raw sewage (untreated wastewater that comes from houses as well as agricultural processes), none of them are concerned about contamination of vegetables and no one is treating the water that they use for the products sanitization.

Concerning the use of protective equipment by farmers, in this study, an observational analysis showed that when they were working in the fields usually did not wear boots and gloves and, the ones that were wearing boots said that it was a form of protection against physical damage and never associated it with occupational diseases.

In the markets, we observed the existence of a gap of knowledge about food safety. Most vendors (60.4%) still believe that most vegetables can cause disease because of the neglected performance time of the pesticides and only a smaller percentage (31.3%) believe that this can be caused by poor hygiene. These results show that vendors still need to be taught about the contamination of fresh food, namely vegetables, about the consequences for human health of that contamination and still need to be trained in good hygiene practices when handling fresh produce.

Another issue to take into consideration to ensure the quality of products, is the way products are transported. The fact that horticultural products are transported in propylene raffia bags and plastic bags, covered and tied, can reduce their freshness and quality. The lack of proper store boxes and the transportation of vegetables with straw bags or traditional ones are typical of developing countries [24]. In general, when vegetables are transported this way, there are high risks of microbiological contamination in addition to chemical and physical risks, especially when using public transportation [25]. These risks can arise due to cross contamination, the environment involved and the dust to which they are exposed. Moreover, climate and sunlight are risky for fresh, green vegetables due to rapid bacterial growth [24]. In fact, in this study, the transport of lettuce in wheelbarrows, wrapped in plastic bags, was observed. Antwi-Agyei et al. [26], showed evidence of the subsequent contamination of vegetables in the markets, when the same products were analyzed before at the farmer and later after transport, confirming the contamination during transportation. Regarding this issue, the “level of education” of the vendors in the different markets had no significant influence (*p* = 0.053, Table 3) in the improvement of their attitudes towards the conditions of transport of fresh produce. Concerning the storage of the fresh vegetables, the fact that none of the markets have an institutional storehouse increases their vulnerability, as insects, possible vectors of pathogenic organisms, can easily access the products and compromise their microbiological safety [27,28]. To keep their products safe, vendors can only rely on themselves and use their own financial and physical resources, which leads many to risky practices in their daily activities. To transport horticultural products in a refrigeration system, a similar structure would be required for display and storage [29,30]. In this study, it was observed that in the Maputo city markets, the hygiene of vegetables can vary between markets, according to the degree of information of vendors regarding good practices in handling fresh produce and the resources available in each market. In the case of the Benfica market, which does not have tap water, most vendors (70%) expressed concern about maintaining the food security of their products and the way they wash them. While in the case of the Fajardo market, which has tap water almost every day, vendors are not concerned with the safety of their vegetables—always washing them in the same plastic containers that are never disinfected. Some authors have reported that the vendors’ main concern is only the attractive presentation of the products rather than their quality, which reflects their misunderstanding of good practices in handling fresh food [31,32,33]. An example of this is the vendors in the Fajardo market, who believe that attractive and shiny products are enough to sell and are not concerned with their food security. Sometimes, the vendors sprinkle water to the vegetables to give them a fresh and attractive look. Such incorrect practices lead to the occurrence of pathogens in fresh produce and the emergence of more cases of diseases associated with them, suggesting the need for tighter vendors’ supervision and their retraining about good health and sanitation practices [26,34]. The comparison of the gender issue with product sanitization showed that women are the ones who best adhere to the attitudes of good practices (*p* = 0.008, Table 3). This finding should also be taken into account when training vendors in the markets.

The sanitization of horticultural products with tap water and sanitizing solution reduces the biological and chemical contaminants from their source [21,35,36]. Meanwhile, the way products are washed and the use of the same containers to wash all vegetables increase the possibility of contamination [37]. To reduce contamination, water must be changed frequently [22] and the containers also need to be disinfected frequently.

From an observational analysis, it was possible to verify that there are markets that benefit daily from tap water, due to their location downtown, where the water supply system works well. On the other hand, there are markets in distant areas of the city, where the water supply system does not work properly and the water supply is seasonal or even sometimes fails, interfering with operability of these markets (cleaning, washing and hygiene actions), thus also interfering with the quality of the daily work of the vendors, as is the case of the Benfica market. In fact, access to tap water in the markets shows a significant threshold difference (*p* = 0.004, see Table S1 in the Supplementary Materials), the Central and Fajardo markets have better conditions of access to water than the rest. The attitude of washing vegetables before sale shows that there is a statistical difference between the markets (*p* = 0.035), were Benfica and Central da Baixa (70% and 80%) had higher rate of washing products before sale, when compared to the Fajardo and Zimpeto markets (28.6% and 31.6%). Regarding the washing system, the results showed that washing vegetables under running water in the markets is more hygienic than washing them in containers, with this difference being statistically significant (*p* = 0.010) (see Table S1 in the Supplementary Materials). The Zimpeto market showed greater adherence to the good vegetables washing practices with tap water than the other markets. The Fajardo market, on the contrary, is the one that most neglects basic hygiene rules, despite having better conditions to do it correctly, access to tap water every day. Barrera et al. considered that for a better sanitization of vegetables, it is necessary to wash them at least twice [38]: the first wash removes the remains of sand or earth and must be done with tap water, while the second wash must be done with a mixture of water and a sanitizing solution to remove organisms. According to another study [32] in rural areas of Sharkyia, Egypt, the use of certain types of disinfectants as potassium permanganate can effectively reduce the transmission of parasitic infections.

The lack of resources in the markets is considered a limitation to their proper functioning in sanitary terms—e.g., sanitary services without tap water for basic hygiene can contribute to the contamination of vegetables by pathogenic microorganisms, namely viruses, bacteria, fungi and parasites. This was one of the factors for the dissemination of microorganisms in a study reported in Ghana [24]. Another study carried out in Maputo shows that the sharing of public toilets, often in poor conditions, and the scarcity of soap in the sanitary facilities increases the risk of contamination due to poor hygiene and maintenance, contributing to the spread of microorganisms [39,40]. Studies in different African countries reported that most public sharing toilets in markets do not have operating conditions, sometimes not even water [39,41]. We noticed that, although some markets have tap water, in Fajardo and sometimes Zimpeto markets, management entities preferred to place the water for general use in containers where vendors can wash their hands with others’ help or even directly into the recipient. Through a univariate analysis, this issue—“*is there water? how is it available?*” showed a highly significant difference (*p* = 0.000) (see Table S1 in the Supplementary Materials) between markets. Central da Baixa and Fajardo were the two markets with good conditions of water access in toilets and tap water availability. The statistical multivariable analysis also proved a significant difference with *p* = 0.020 (Table 3) when we analyzed the availability of resources in the markets (water and toilets). These resources are limiting factors for the correct attitude of vendors towards their personal hygiene and can interfere with the quality of the products that they offer for sale if they do not have basic conditions or resources for adequate hygiene. These limiting resources are also related to the availability of adequate physical facilities, which exist, for example, in the old Central da Baixa market in Maputo but do not exist in the formal and informal peri-urban markets that can appear spontaneously.

Another factor that can interfere with the quality of products is the waste management in each sector of horticultural products sold. PSAS has different solutions for waste management and collection; however, they are not as efficient as those used throughout the city. In outer suburban areas, there is no municipal waste collection; therefore, the garbage must be burned, buried or used for animal feed. For this reason, the waste management in such areas is difficult, as are their associated health problems. Studies in Maputo city emphasized the deficient waste management, lack of sanitation and solid waste management system, especially in peri-urban areas, due to the deficit in containers and reduced public environmental awareness [17,42]. According to municipal entities, the cleaning of the markets is the vendors’ entire responsibility in each vending stand, but the Municipal City Council is responsible for the waste collection all over the city, via the PGRSS department. This department is not acting alone, but in association with private companies that help collect waste for the municipal disposal site. According to the city council, the vendors’ commissions in each market should make an effort to control their work and avoid the accumulation of garbage in the markets. This attitude and concern are part of their obligations. Although the vendors’ responsibility must be taken into account, management entities must also ensure the efficiency of their collaborative work.

In all markets analyzed—the five most popular in the city of Maputo—it was found that there was weak supervision of the vendors’ activities by the management entities, mainly by the City Council. The PMF of the City Council of Maputo is responsible for selecting the inspectors in each market and these ones must manage their commercial establishment in accordance with the regulations of the markets and fairs. They are the bridge of communication between PMF and vendors through the vendors’ commission. Besides, the City Council has an essential collaborator, PSAS, which assists in campaigns for sanitization of environment, cleaning, and supervision, in addition to working on social problems for each market. However, the collaboration of PSAS in problem solving is limited because existing regulations are not punitive. In cases where corrective actions are required, PSAS acts only by giving the vendor specific time to correct its posture, according to the infraction, then returning later to supervise the product or the commercial establishment. In return, any vendor or consumer can complain about the abnormal PSAS actions. Nowadays, vendors take advantage of conflicts between PSAS and INAE to not allow PSAS to supervise their commercial establishments, and this represents another problem for the entities to guarantee efficient work. To avoid such situations, it is urgent to decide which entity is responsible for ensuring the supervision of products and the quality of commercial establishments, as well as the limits of their intervention.

## 5. Conclusions

In Maputo, Mozambique, markets are the main supply of horticultural products to populations, which can be a challenge for public health authorities due to the risk for the population to acquire gut pathogens. In this study, we hypothesize that the transmission of these organisms may be associated with inadequate product transportation from the farm to the market, and the poor sanitation in the markets, due to lack of water resources, lack of good toilets conditions and vendors’ low health-risk perception. Last but not least, it may also be associated with the use of wastewater to irrigate cultivated fields around the peri-urban area of Maputo.

To help overcome this health threat, policies on market operating regimes must be clearer and the technical staff must have full knowledge of these rules; otherwise, there will be greater difficulty in implementing and monitoring actions with vendors, as is currently the case, due to conflicts between PSAS and INAE. The PMF should also consider creating regulations for the sale of vegetables in farmers’ fields, because they are the first major suppliers of vegetables in Maputo City, especially the leafy ones. Clean and disinfected water for washing fresh vegetables is fundamental at all stages of the vegetable supply chain, from the production fields to the vendors in the markets, before buying/selling these products. In this study, we strongly recommend that seasonal vendors make regular use of good hygiene and agricultural practices, emphasizing the good influence of women, as we have seen, they were the ones who presented the best practical assimilation in these areas. We also recommend regular supervision of the technical team’s fieldwork by the city council. Only with strict supervision measures will it be possible to reduce the risk, for buyers/consumers, of acquiring a gastrointestinal infection due to the purchase of contaminated fresh produce. Further studies need to be implemented in other fresh vegetables markets of Maputo city, and updates should be made at least every four years to the general regulations of the fairs and markets for the better supervision of the products.

## Figures and Tables

**Table 1:** Profile of the vendors of the five markets studied.

Variables	Markets					
	Benfica N/%	Xipamanine N/%	Central da Baixa N/%	Zimpeto N/%	Fajardo N/%	Total N/%
Gender						
Male	2/10%	1/8%	2/20%	5/26%	0/0%	10/13.3%
Female	18/90%	11/92%	8/80%	14/74%	14/100%	65/86.7%
Total	20/27%	12/16%	10/13%	19/25%	14/19%	75/100%
Age						
< 18	1/5%	**0/0%**	0/0%	0/0%	1/7%	2/2.7%
18–45	12/60%	4/33%	3/30%	15/79%	4/29%	38/50.7%
> 45	7/35%	8/67%	7/70%	4/21%	9/64%	35/46.7%
Total	20/27%	12/16%	10/100%	19/25%	14/19%	75/100%
Education						
Iliterate	1/5%	4/33%	0/0%	3/16%	2/14%	10/13.3%
Primary	9/45%	**5/42%**	4/40%	8/42%	8/57%	34/45.3
Secondary	10/50%	3/25%	6/60%	8/42%	4/29%	31/41.3
Total	20/27%	12/16%	10/13%	19/25%	14/19%	75/100%

**Table 2:** Origin of production and supply chain of horticultural products in the five markets studied.

Origin of Production			Markets			
	Benfica N/%	Xipamanine N/%	Central da Baixa N/%	Zimpeto N/%	Fajardo N/%	Total (%)
Green zones *	9/45%	4/33%	1/10%	5/26%	4/29%	23/31%
Wholesale markets	11/55%	6/50%	3/30%	4/21%	9/64%	33/44%
Retailers markets	0/0%	2/17%	5/50%	0/0%	1/7%	8/15%
Outside the city	0/0%	0/0%	1/10%	10/53%	0/0%	11/15%
Total	20/27%	12/16%	10/13%	19/25%	14/19%	75/100%

* “Zonas verdes/Green zones” or “Cintura verde/Green belt”—The two big areas where horticultural products are cultivated, mostly leafy horticultural products, in peri-urban areas of Maputo City. They are Mahotas area and the Infulene valley and are supplied by the drainage ditch of the Infulene and Mulaúze rivers.

**Table 3:** Multivariable analysis comparing vendor’s profile (age, gender and education) against their knowledge of good hygiene practices and sanitization and comparison of markets location and distribution of management resources availability.

Dependent Variables	Independent Variables	Unstandardized Coefficients B	Standard Error	Standardized Coef. (Beta)	t	Sig. (*p* Value)	R Square Adjusted	Sig ANOVA
Knowledge attitudes	(Constant)	2,295	1,467		1.565	0.122	0.019	0.228
	Age	0.298	0.245	0.15	1.215	0.228		
	Gender	−0.124	0.601	−0.024	−0.207	0.837		
	Education	0.604	0.306	0.239	1.972	0.053		
Sanitization	(Constant)	4.255	0.924		4.607	0.000	0.087	0.024
	Age	0.156	0.154	0.121	1.009	0.316		
	Gender	−1.027	0.378	−0.309	−2.713	0.008		
	Education	0.157	0.193	0.095	0.815	0.418		
Location of the markets	(Constant)	0.532	1.004		0.530	0.598	0.058	0.044
	“*Is there any toilet near*?”	0.230	0.244	0.106	0.942	0.350		
	“*Is there water? how is it available*?”	0.556	0.235	0.267	2.363	0.021		

## Supplementary Materials

The following are available online at https://www.mdpi.com/1660-4601/17/17/6302/s1, Table S1: Resume of vendors’ attitude and their knowledge on Good Hygiene Practices.
