# Supplementary material for: Handling of Fresh Vegetables: Knowledge, Hygienic Behavior of Vendors, Public Health in Maputo Markets, Mozambique
Source: Int J Environ Res Public Health. 2020 Aug 29;17(17):6302. doi: 10.3390/ijerph17176302 (PMC7504209; doi:10.3390/ijerph17176302)
Supplement: Supplementary file 1 [file ijerph-17-06302-s001.pdf]

Supplementary Materials

Table S1 – Resume of vendors' attitude and their knowledge on Good Hygiene Practices.

VARIABLES | *MARKETS*

ACESS TO TAP WATER	**BENFICA N/%**	**XIPAMANINE N/%**	**CENTRAL DA BAIXA N/%**	**ZIMPETO N/%**	**FAJARDO N/%**	**TOTAL N/%**	***P* VALUE**
YES	16/80.0%	6/50.0%	10/100%	17/89.0%	14/100%	63/84.0%	
NO	4/20.0%	6/50.0%	0/0%	2/11.0%	0/0%	12/16.0%	
TOTAL						75/100%	0.004
WASHING PRODUCTS BEFORE SALE	**BENFICA**	**XIPAMANINE**	**CENTRAL DA BAIXA**	**ZIMPETO**	**FAJARDO**	**TOTAL N/%**	***P* VALUE**
YES	14/70.0%	8/66.7%	8/80.0%	6/31.6%	4/28.6%	40/53.3%	
NO	6/30.0%	4/33.3%	2/20.0%	13/68.4%	10/71.4%	35/46.7%	
TOTAL					14/19%	75/100%	0.035
HOW DO YOU WASH THEM?	**BENFICA**	**XIPAMANINE**	**CENTRAL DA BAIXA**	**ZIMPETO**	**FAJARDO**	**TOTAL N/%**	***P* VALUE**
RUNNING WATER	0/0%	0/0%	1/12.5%	2/33.3%	0/0%	3/7.5%	
IN RECIPIENTS (BUCKETS AND GALLONS)	14/100%	8/100%	7/87.5%	4/66.7	4/100%	37/92.5%	
TOTAL						40/100%	0.010
DO YOU THINK VEGETABLES CAN CAUSE DISEASE?	**BENFICA**	**XIPAMANINE**	**CENTRAL DA BAIXA**	**ZIMPETO**	**FAJARDO**	**TOTAL N/%**	***P* VALUE**
YES	14/70.0%	8/67.0%	6/60.0%	12/63.0%	8/57.0%	48/64.0%	
NO	6/30.0%	2/17.0%	3/30.0%	7/37.0%	2/14.0%	20/26.7%	
CAN NOT SAY	0/0%	2/17.0%	1/10.0%	0/0%	4/29.0%	7/9.3%	
						75/100%	0.164
WHY DO YOU THINK VEGETABLES CAN CAUSE DISEASE?	**BENFICA**	**XIPAMANINE**	**CENTRAL DA BAIXA**	**ZIMPETO**	**FAJARDO**	**TOTAL N/%**	***P* VALUE**
LACK OF HYGIENE	5/35.7%	1/12.5%	4/66.7%	4/33.3%	1/12.5%	15/31.3%	
PESTICIDES	6/42.9%	7/87.5%	2/33.3%	8/66.7%	6/75.0%	29/60.4%	
PESTICIDES & LACK OF HYGIENE	2/14.3%	0/0%	0/0%	0/0%	1/12.5%	3/6.3%	
CAN NOT SAY	1/7.1%	0/0%	0/0%	0/0%	0/0%	1/2.1%	
TOTAL						48/100%	0.611
HOW DO YOU TRANSPORT THE PRODUCTS?	**BENFICA**	**XIPAMANINE**	**CENTRAL DA BAIXA**	**ZIMPETO**	**FAJARDO**	**TOTAL N/%**	***P* VALUE**
OPEN-TRUCK VEHICLE	7/35.0%	9/75.0%	5/50.0%	16/84,2%	7/50.0%	44/58.7%	
TCHOVA	4/20.0%	0/0%	0/0%	2/10.5%	0/0%	6/8.0%	
PUBLIC TRANSPORT	1/5.0%	2/16.7%	2/20.0%	0/0%	0/0%	5/6.7%	
ON FOOT	1/5.0%	1/8.3%	0/0%	1/5.3%	1/7.1%	4/5.3%	
OPEN-TRUCK VEHICLE AND PUBLIC TRANSPORT	7/35.0%	0/0%	3/30.0%	0/0%	6/42.9%	16/21.3%	
TOTAL						75/100%	0.014
WHERE DO YOU STORE THE	**BENFICA**	**XIPAMANINE**	**CENTRAL DA BAIXA**	**ZIMPETO**	**FAJARDO**	**TOTAL N/%**	***P* VALUE**

PRODUCTS IN THE END OF THE DAY?							
AT HOME	4/20.0%	1/8.3%	0/0%	5/26.3%	3/21.4%	13/17.3%	
PRIVATE STOREHOUSE OUTSIDE THE MARKET	9/45.0%	1/8.3%	0/0%	0/0%	6/42.9%	16/21.3%	
PRIVATE STOREHOUSE INSIDE THE MARKET	2/10.0%	5/41.7%	0/0%	8/42.1%	2/14.3%	17/22.7%	
BELOW THE STAND IN THE MARKET	5/25.0%	5/41.7%	10/100%	6/31.6%	3/21.4%	29/38.7%	
TOTAL						75/100%	0.000
IS THERE ANY TOILET NEAR?	**BENFICA**	**XIPAMANINE**	**CENTRAL DA BAIXA**	**ZIMPETO**	**FAJARDO**	**TOTAL N/%**	***P* VALUE**
YES, FROM CITY COUNCIL (CC)	16/80.0%	11/91.7%	10/100%	16/84.2%	9/64.3%	62/82.7%	
YES, PRIVATE	3/15.0%	1/8.3%	0/0%	3/15.8%	4/28.6%	11/14.7%	
YES, FROM CC AND PRIVATE	1/5.0%	0/0%	0/0%	0/0%	1/7.1%	2/2.7%	
TOTAL						75/100%	0.506
IS THERE WATER? HOW IS IT AVAILABLE?	**BENFICA**	**XIPAMANINE**	**CENTRAL DA BAIXA**	**ZIMPETO**	**FAJARDO**	**TOTAL N/%**	***P* VALUE**
YES, TAP WATER	1/5.0%	0/0%	10/100%	8/42.1%	7/50.0%	26/34.7%	
YES, IN RECIPIENTS	17/85.0%	7/58.3%	0/0%	10/52.6%	6/42.9%	40/52.3%	
SOMETIMES IN RECIPIENTS	1/5.0%	3/25%	0/0%	1/5.3%	1/7.1%	6/8.0%	
NO	1/5.0%	2/16.7%	0/0%	0/0%	0/0%	3/4.0%	
TOTAL						75/100%	0.000
HOW IS THE GARBAGE COLLECTION?	**BENFICA**	**XIPAMANINE**	**CENTRAL DA BAIXA**	**ZIMPETO**	**FAJARDO**	**TOTAL N/%**	***P* VALUE**
IN TRASH BAGS AND AFTER DUMP IN THE CONTAINERS	18/90.0%	12/100%	6/60.0%	17/89.5%	9/64.3%	62/82.7%	
DIRECTLY TO THE CONTAINERS	1/5.0%	0/0%	4/40.0%	2/10.5%	4/28.6%	11/14.7%	
BOTH TYPES	1/5.0%	0/0%	0/0%	0/0%	1/7.1%	2/2.7%	
TOTAL						75/100%	0.084
